# SARS-CoV-2 Aerosol and Intranasal Exposure Models in Ferrets

**DOI:** 10.3390/v15122341

**Published:** 2023-11-29

**Authors:** Elizabeth E. Zumbrun, Samantha E. Zak, Eric D. Lee, Philip A. Bowling, Sara I. Ruiz, Xiankun Zeng, Jeffrey W. Koehler, Korey L. Delp, Russel R. Bakken, Shannon S. Hentschel, Holly A. Bloomfield, Keersten M. Ricks, Tamara L. Clements, April M. Babka, John M. Dye, Andrew S. Herbert

**Affiliations:** 1 Division of Virology, United States Army Medical Research Institute of Infectious Disease, Frederick, MD 21702, USA; samantha.e.zak.ctr@health.mil (S.E.Z.); russel.r.bakken.civ@health.mil (R.R.B.); shannon.s.hentschel2.ctr@health.mil (S.S.H.); john.m.dye1.civ@health.mil (J.M.D.); andrew.s.herbert4.civ@health.mil (A.S.H.); 2 Division of Pathology, United States Army Medical Research Institute of Infectious Disease, Frederick, MD 21702, USA; eric.d.lee2@gmail.com (E.D.L.); xiankun.zeng.civ@health.mil (X.Z.); holly.a.bloomfield.civ@health.mil (H.A.B.); april.m.babka.civ@health.mil (A.M.B.); 3 Division of Veterinary Medicine, United States Army Medical Research Institute of Infectious Disease, Frederick, MD 21702, USA; philip.bowling.1@us.af.mil; 4 Division of Bacteriology, United States Army Medical Research Institute of Infectious Disease, Frederick, MD 21702, USA; sara.i.ruiz.civ@health.mil; 5 Diagnostic Systems Division, United States Army Medical Research Institute of Infectious Disease, Frederick, MD 21702, USA; jeffrey.w.koehler4.civ@health.mil (J.W.K.); korey.l.delp.ctr@health.mil (K.L.D.); keersten.m.ricks.civ@health.mil (K.M.R.); tamara.l.clements.civ@health.mil (T.L.C.)

**Keywords:** SARS-CoV-1, coronavirus, COVID, COVID-19, ferret, animal model, pathogenesis, intranasal, aerosol, small particle

## Abstract

Severe acute respiratory syndrome coronavirus 2 (SARS-CoV-2) is the causative agent of the worldwide COVID-19 pandemic. Animal models are extremely helpful for testing vaccines and therapeutics and for dissecting the viral and host factors that contribute to disease severity and transmissibility. Here, we report the assessment and comparison of intranasal and small particle (~3 µm) aerosol SARS-CoV-2 exposure in ferrets. The primary endpoints for analysis were clinical signs of disease, recovery of the virus in the upper respiratory tract, and the severity of damage within the respiratory tract. This work demonstrated that ferrets were productively infected with SARS-CoV-2 following either intranasal or small particle aerosol exposure. SARS-CoV-2 infection of ferrets resulted in an asymptomatic disease course following either intranasal or small particle aerosol exposure, with no clinical signs, significant weight loss, or fever. In both aerosol and intranasal ferret models, SARS-CoV-2 replication, viral genomes, and viral antigens were detected within the upper respiratory tract, with little to no viral material detected in the lungs. The ferrets exhibited a specific IgG immune response to the SARS-CoV-2 full spike protein. Mild pathological findings included inflammation, necrosis, and edema within nasal turbinates, which correlated to positive immunohistochemical staining for the SARS-CoV-2 virus. Environmental sampling was performed following intranasal exposure of ferrets, and SARS-CoV-2 genomic material was detected on the feeders and nesting areas from days 2–10 post-exposure. We conclude that both intranasal and small particle aerosol ferret models displayed measurable parameters that could be utilized for future studies, including transmission studies and testing SARS-CoV-2 vaccines and therapeutics.

## 1. Introduction

Since its emergence in 2019, severe acute respiratory syndrome coronavirus 2 (SARS-CoV-2) has infected over 750 million individuals worldwide, leading to more than 6.8 million deaths [1,2]. The earliest known human case can be traced back to Wuhan City, Hubei Province, China, in December 2019, and a global pandemic was declared by the WHO in March 2020 [3]. SARS-CoV-2 is spread from human to human predominantly via respiratory droplets or fomite transmission [4]. The primary route of transmission via droplet is facilitated by the affinity of SARS-CoV-2 to replicate within the upper respiratory tract [3]. The clinical signs of disease can range from asymptomatic to severe, with the older population and those with underlying conditions being disproportionally impacted [5]. In symptomatic patients, fever is the most common symptom (92.8%), followed by cough (69.8%), dyspnea (34.5%), and myalgia (27.7%) [5]. Other symptoms can include headache, diarrhea, rhinorrhea, and sore throat [6]. While disease in the lung is variable, pneumonia with bi-lateral lung involvement and life-threatening systemic inflammatory disease such as acute respiratory distress (ARDS), myocardial inflammation, and multi-organ dysfunction can occur in severe cases [3]. In contrast, many cases are asymptomatic but can nonetheless efficiently spread due to the ability of the virus to propagate within the oropharyngeal regions during early infection [3,7].

SARS-CoV-2 is a positive-sense single-stranded RNA virus in the genus β-coronavirus with 88–89% similarity to two bat-derived SARS-like coronaviruses (bat-SL-CoVZC45 and bat-SL-CoVZXC21) [8]. SARS-CoV-2 primarily utilizes the host angiotensin converting enzyme II (ACE2) receptor via the viral spike (S) glycoprotein for cell entry that is composed of two subunits (S1 and S2) [9,10,11]. SARS-CoV-2 S has a high degree of similarity (99%) to SARS-CoV S2 and a 70% similarity to the SARS-CoV S1 protein at the amino acid level [10]. The genome of SARS-CoV-2 is 79–82% similar to SARS-CoV and 50% similar to Middle East respiratory syndrome coronavirus, both of which have been previously studied in small animal models and non-human primates (NHPs) [12,13]. 

As evidenced by years of influenza research, ferrets (*Mustela putorius furo*) are a useful animal model for respiratory viruses due in part to the similarities between ferret and human respiratory tracts [14]. Research on SARS-CoV pointed to the possibility of several robust small animal model options that could be pursued for SARS-CoV-2 [15]. SARS-CoV infection of ferrets, primarily by the intranasal and intratracheal routes, resulted in a productive infection of the lungs, trachea, and nasal turbinates with viral replication peaking at around 6 days post-exposure (dpe) [16,17]. Pathological findings included infiltration of lymphocytes and macrophages into the lungs, mild alveolar damage, and pulmonary lesions [16]. The clinical signs included fever, sneezing, lethargy, and some prolonged disease. Mortality was a rare event across studies. The experimental design herein is based on findings from research conducted on SARS-CoV using the ferret model [16,17,18,19,20,21,22,23]. This model, applied to SARS-CoV-2, is useful not only for the study of the disease and screening of countermeasures [24,25] but also for investigating the pathogenesis [26] and transmission differences among variants [27,28,29]. 

Previously published SARS-CoV-2 research in ferrets has predominantly utilized the intranasal route of challenge, although ocular and intratracheal infections have also been explored [15,24,25,29,30,31,32,33,34,35] and reviewed [36,37,38,39]. These ferret models have been utilized to evaluate vaccine [24,25,40] and therapeutic [41,42,43,44,45,46] candidates for SARS-CoV-2. For example, Boley et al. utilized the IN SARS-CoV-2 ferret model to explore intranasal lipid nanoparticle-based SARS-CoV-2 protein and mRNA-based vaccines [24]. Additionally, Martins et al. used the IN SARS-CoV-2 model to demonstrate that a DNA vaccine candidate reduced viral shedding [25]. Finally, Cox et al. used the ferret IN the SARS-CoV-2 model to look at the impact of therapeutics on transmission [29].

Ferret models have also been used to demonstrate transmission via direct or indirect contact and airborne transmission [3,15,28,29,30,47,48,49,50,51,52]. This line of study is important because aerosols play an important role in transmission among humans [53,54,55,56]. SARS-CoV-2 is viable in aerosols for at least 3 h, with small particles staying airborne indefinitely [55,57,58]. Aerosols vary in size and can be generated from breathing, coughing, speaking, and sneezing. The majority of aerosol particles generated from breathing [59], coughing [60], and speaking [55] are mostly very small (<2 µm, <1 µm, and ~4 µm, respectively), whereas the aerosol particles from sneezes are large with peaks at approximately 72 µm and 386.2 µm [61]. As such, small and large particle aerosols may more accurately mimic a human-to-human transmission event, produce a more clinically relevant delivery to the airways, and result in a more consistent disease model. 

James et al. explored large particle (4–106 µm) aerosol exposure of SARS-CoV-2 in ferrets as compared to intranasal exposure [62]. Larger particles greater than 8 µm primarily deposit in the nasal passages and larger bronchioles of the lung [63]. Ferrets were exposed to the large particle aerosols generated by a mucosal atomization device for approximately one minute and did not become robustly infected or seroconvert. Only low levels of SARS-CoV-2 viral RNA were detected in nasal washes of aerosol-exposed ferrets compared to those exposed by the intranasal route. 

Our study is the first to assess small particle (~3 µm) aerosol exposure in ferrets, which is important since the majority of particles generated by breathing, coughing, and talking are small particles that have a greater potential to travel beyond the upper respiratory tract and diffuse into the deep lung, depositing in the alveoli. We explored both intranasal and small particle aerosol routes of delivery. Target doses of at least 10^3^–10^5^ pfu were proposed for the inoculation based on the SARS-CoV and SARS-CoV-2 literature available at the time this study was conducted [16,18,64,65,66,67]. Measurements following SARS-CoV-2 exposure of ferrets included the following parameters: clinical disease score, body weight, temperature, RT-PCR of pharyngeal swabs, RT-PCR (viral genomic and viral subgenomic RNA) of nasal washes, serum IgG assessment by MAGPIX, histopathologic evaluation, immunohistochemistry of upper and lower respiratory tissues, and immunofluorescence staining in lung tissue to detect immune cell infiltration.

## 2. Methods

### 2.1. Animals and Experimental Design

Twenty-four male-specific pathogen (influenza, coccidian, giardia, and ear mite)-free ferrets (*Mustela putorius furo*), aged 20–32 weeks and weighing 1.3–1.8 kg, were purchased from Marshall Bioresources. Ferrets were ear-tagged and shipped to USAMRIID in filtered crates. Animals were acclimated to ABSL-3 for at least 7 days. Animals were weighed and implanted subcutaneously with IPTT-300 temperature/ID microchips (BMDS) prior to the start of the study (day -7). On day 0, animals were exposed to SARS-CoV-2 via the intranasal route (n = 12 per group) or the small particle aerosol route (n = 12 per group). For intranasal exposure, SARS-CoV-2 inoculum was appropriately diluted in PBS (Sigma-Aldrich, St. Louis, MO, USA), and 250 µL was instilled into each nare (total volume of 500 µL) of anesthetized animals. For exposure to small particle (~3 µm) aerosol, unanesthetized animals were placed within a wire mesh cage in a whole-body aerosol chamber. The SARS-CoV-2 target aerosol exposure dose was based on the weight of each animal using Guyton’s formula. Up to four animals were exposed simultaneously.

On the day of challenge, temperatures and weights were collected, animals were anesthetized, and blood and pharyngeal swabs were collected prior to virus exposure. Nasal washes and pharyngeal swabs were also collected under anesthesia on days 2, 4, 7, 10 and 14 post-exposure for analysis of viral load by RT-PCR. On days 4, 7, 10 and 14, three animals from each group were euthanized, and terminal blood collection and necropsy for collection of tissues were conducted. During the baseline and in-life periods, animals were observed for clinical signs of disease, and temperatures and body weights were taken once per day at approximately the same time each day and scored as described in the “Observations” method section below. Historical unexposed ferret lung tissues were used as controls for immunofluorescence staining.

### 2.2. Challenge Agent

A seed stock of SARS-CoV-2, Washington State’s first isolate in 2020 (WA-1/2020), designated as Lot R4717, accession number MW925130.1, was grown on ATCC Vero 76 cells. The seed stock contains an average of 1.56 × 10^6^ pfu/mL using an agarose and neutral red-based plaque assay. R4717 was fully sequenced, evaluated for sterility, tested for mycoplasma and endotoxin levels, and tested in a number of real-time reverse transcriptase polymerase chain reaction (RT-PCR) assays to include two specific for SARS-CoV-2 virus. The SARS-CoV-2 stock contained complete Minimum Essential Medium (Corning Inc., Corning, NY, USA) containing 5% fetal calf/bovine serum (FBS) (Hyclone, Logan, UT, USA) and was stored at −60 to −90 °C.

For challenge (day 0), the challenge agent was prepared using neat stock to achieve the maximum possible dose for intranasal and aerosol administration. An aliquot of pooled SARS-CoV-2 master seed stock was assessed using a standard agarose (Lonza, Walkersville, MD, USA) and neutral red staining plaque assay on ATCC Vero 76 cells. Briefly, required dilutions of each specimen (starting concentration at 10^−1^ through 10^−6^ for intranasal challenge inoculum, and all glass impingers at 10^−1^ through 10^−4^), in triplicate, were added to plates containing ATCC Vero 76 cells [68]. Two days later, the cells were stained with neutral red, and plaques were counted the next day. Titers were based on the most concentrated dilution series with mean plaque counts between 10 and 150.

### 2.3. Observations

All animals were observed daily for the 7-day baseline acclimation period preceding the challenge (day 0). After the challenge, all animals were observed daily. All ferrets were exposed to SARS-CoV-2 either via IN or small particle AE with no unscheduled deaths or euthanasia events. Scheduled necropsies were conducted at 4, 7, 10 and 14 dpe. Therefore, neither IN nor AE exposure of ferrets to SARS-CoV-2 resulted in death during the timeframe of this study.

Daily observations were performed in accordance with the study schedule from day -7 onward. During the baseline and in-life periods, animals were observed for clinical signs of disease at approximately the same time each day. Observations included assessment of general behavior, sneezing, respiratory signs, ocular or nasal discharge, diarrhea, and any other clinical signs. Behavior was scored as follows: 0 = minor change, less active, or subdued but normal when stimulated; 3 = little activity, less mobile/alert, subdued when stimulated; and 7 = still, vocalization, self-mutilation, or no reaction to stimulus. Additionally, temperatures and body weights were taken once per day at the time of the observation. Body weights were scored as follows: 0 = less than 5% weight loss or gain; 1 = 5% and up to 10% weight loss from baseline; 3 = 10% and up to 20% weight loss from baseline; and 7 = greater than 20% weight loss from baseline.

### 2.4. Specimen Collection

#### 2.4.1. Blood Collection

Blood was collected for laboratory assays at scheduled euthanasia events at 4, 7, 10 and 14 dpe. Blood volumes did not exceed the limits described in USAMRIID standard operating procedures and the IACUC protocol. Two TRIzol LS (Thermo Fisher Scientific, Waltham, MA, USA) aliquots and up to four no-additive aliquots were prepared. 

#### 2.4.2. Pharyngeal Swab Collection and Processing

Pharyngeal swabs were collected into a 15 mL conical tube containing 1.0 mL of viral transport media (VTM; Hanks Balanced Salt Solution containing 2% heat-inactivated fetal bovine serum, 100 µg/mL gentamicin, and 0.5 µg/mL amphotericin B) (Thermo Fisher Scientific, Waltham, MA, USA).

Pharyngeal swab specimens were vortexed for 15–20 s and then incubated at 2–8 °C for 20–25 min. Following incubation, specimens were again vortexed for 15–20 s. The swab suspension was then moved into a new sterile tube for clarification. These samples were clarified using a centrifuge set at 14,000 rpm for 30 s. Clarified lysate was removed from the pellet within 10 min of centrifugation. Two TRIzol LS aliquots (100 µL sample plus 300 µL TRIzol) and two no-additive clarified lysate aliquots of up to 400 µL were prepared and stored at −60 to −90 °C.

#### 2.4.3. Nasal Wash Collection and Processing

Nasal washes were conducted with 2.0 mL of PBS administered to the nares with a syringe and collected into a 15 mL conical tube. Nasal wash samples were moved into a new sterile 2 mL tube for clarification. These samples were clarified using a centrifuge set at 14,000 rpm for 30 s. Clarified lysate was removed from the pellet within 10 min of centrifugation. Two TRIzol LS aliquots (100 µL sample plus 300 µL TRIzol and two no-additive clarified lysate aliquots of up to 800 µL were prepared and stored at −60 to −90 °C. 

### 2.5. Detection of Viral RNA by Real-Time RT-PCR

Serum and clarified swab specimens were inactivated with TRIzol LS in a ratio of 3 parts TRIzol to 1 part sample. Inactivated specimens were then extracted and eluted with AVE buffer using a QIAamp Viral RNA Mini Kit (Qiagen, Hilden, Germany). The RT-PCR reaction utilized Invitrogen™ SuperScript One-Step RT-PCR System with additional magnesium sulfate (MgSO_4_) added to a final concentration of 3.0 mM (Thermo Fisher Scientific, Waltham, MA, USA). Specimens were run in triplicate using a 5 µL volume. The average of the triplicates was multiplied by 200 to obtain target copies per mL, and then multiplied by a dilution factor of 4 (1 part sample to 3 parts TRIzol LS) for the final reported value. The genomic equivalents (ge) were determined using a standard curve of synthetic RNA of known concentration. The Applied Biosystems 7500 Fast Dx instrument was used to run the samples. The lower limit of quantitation (LLOQ) of the assays were as follows: N2 = 7.7 Log_10_ copies/mL (CT = 36.63), GP = CT = 36.49, and E = 5 Log_10_ copies/mL (CT = 34.15). A CT cutoff value of 40 was used for each assay and was considered the limit of detection (LOD) and corresponds to the following values: N2 = 3.03 log_10_ copies/mL and N2 = 3.03 log_10_ copies/mL. Samples with no signal are plotted as ½ the LOD (2.73 log_10_ copies/mL and 2.81 log_10_ copies/mL for N2 and E assays, respectively), or at CT = 41 (for the G assay).

### 2.6. Subgenomic Viral RNA Expression by RT-PCR 

Clarified ferret nasal wash samples were inactivated using a 3:1 TRIzol LS Reagent, and total nucleic acid was extracted using the EZ1 Virus Mini Kit v2.0 (Qiagen, Hilden, Germany) and the EZ1 Advanced XL robot (Qiagen, Hilden, Germany) according to the manufacturer’s recommendations. Target copy numbers of total E RNA and subgenomic E RNA for SARS-CoV-2 were determined using a pair of previously described real-time RT-PCR assays [4,69] and a synthetic RNA (Bio-Synthesis, Lewisville, TX, USA) corresponding to the subgenomic E RNA amplicon sequence. 

Samples were run in triplicate (5 μL extracted nucleic acid) for each assay using a Superscript III one-step RT-PCR system with Platinum Taq (Thermo Fisher Scientific, Waltham, MA, USA) and the LightCycler 480 (Roche, Basel, Switzerland). Cycling conditions were 50 °C for 10 min; 95 °C for 3 min; 45 cycles of 95 °C for 10 s, 56 °C for 15 s, and 72 °C for 5 s; and a final hold of 40 °C for 30 s. Copy numbers for each target were determined using the synthetic RNA standard curve, and the amount of target amplicon in the original wash sample was calculated from these results. The LLOQ of this assay is 5 Log_10_ copies/mL (CT = 38.48). A CT cutoff value of 40 was used for what is considered the LOD, corresponding to 4.35 log_10_ copies/mL. Samples with no signal are plotted as ½ the LOD (4.05 log_10_ copies/mL).

### 2.7. SARS-CoV-2 MAGPIX Multiplex Immunoassay

#### 2.7.1. Magnetic Microsphere Production

Recombinant SARS-CoV-2 full trimeric spike (produced from the full spike construct from Jason McLellan’s group; UT-Austin [70]) and NP (Native Antigen Company, Kidlington, UK; REC31812-100) proteins were conjugated to magnetic microspheres using the Luminex xMAP® antibody coupling kit (Luminex Inc., Austin, TX, USA) according to the manufacturer’s instructions. Briefly, 500 µL of Magplex microspheres (DiaSorin, Saluggia, Italy) (12.5 × 10^6^ microspheres/mL) were washed three times using a magnetic microcentrifuge tube holder and resuspended with 480 µL of activation buffer. Then, 10 µL of both sulfo-N-hydroxysulfosuccinimide (sulfo-NHS) (Thermo Fisher Scientific, Waltham, MA, USA) and 1-Ethyl-3-[3-dimethylaminopropyl]carbodiimide hydrochloride (EDC) solutions were added. The tube was covered with aluminum foil and placed on a benchtop rotating mixer for 20 min. After surface activation with EDC, the microspheres were washed three times with activation buffer prior to adding the recombinant protein antigen at a final concentration of 4 µg antigen/1 × 10^6^ microspheres. This concentration of recombinant protein coupled to the surface of microspheres has been shown to be optimal for IgG and IgM detection [71]. The tube was again covered with aluminum foil and placed on a benchtop rotating mixer for 2 h. After this coupling step, the microspheres were washed three times with wash buffer and resuspended in 500 µL of wash buffer for further use. SARS-CoV-2 full spike and NP were coupled to Magplex microsphere regions #45 and #25 (Luminex Inc., Austin, TX, USA), respectively, to facilitate multiplexing experiments. Beads were stored at 4 °C until further use.

#### 2.7.2. Screening SARS-CoV-2 Ferret Serum

Serum samples were diluted 1:100 in phosphate-buffered saline (PBS) with 0.02% Tween-20 (PBST) (Sigma-Aldrich, St. Louis, MO, USA) with 5% skim milk (PBST-SK) (Sigma-Aldrich, St. Louis, MO, USA). Each antigen-coupled bead was mixed with a 1:1 ratio prior to diluting in PBST to 5 × 10^4^ microspheres/mL and added to a Costar polystyrene 96-well plate (Corning Inc., Corning, NY, USA) at 50 µL per well (2500 microspheres of each antigen bead set/well). The plate was placed on a magnetic plate separator (Luminex Inc., Austin, TX, USA) covered with foil, and microspheres were allowed to collect for 60 s. While still attached to the magnet, the buffer was removed from the plate. A total of 50 µL of diluted serum samples was added. The plate was covered with a black vinyl plate cover and incubated with shaking for 1 h at RT. The plate was washed three times with 100 µL of PBST, using the plate magnet to retain the Magplex microspheres in the wells. In addition, 50 µL of a 1:100 dilution of goat-anti ferret IgG (Novus Biologicals LLC, Centennial, CO, USA; NB7222) in PBST-SK was added to the wells. The plate was covered again and incubated with shaking for 1 h at RT. After incubation, the plate was washed three times, as detailed above, prior to adding 50 µL of a 1:100 dilution of donkey anti-goat IgG-PE conjugate (Abcam, Waltham, MA, USA; ab7004) in PBST-SK and incubated with shaking for 1 h at RT. The plate was washed three times, and the Magplex microspheres were resuspended in 100 µL of PBST for analysis on the Magpix instrument. Raw data was reported as median fluorescence intensity for each bead set in the multiplex. 

### 2.8. Terminal Procedures and Anatomic Pathology

#### 2.8.1. Termination

Euthanasia was performed in accordance with the procedures described in the IACUC protocol. Three animals per group were euthanized at 4, 7, 10 and 14 dpe.

#### 2.8.2. Anatomic Pathology

##### Gross Necropsy

A veterinary pathologist conducted necropsies on all study animal carcasses in the containment suite. All gross findings were recorded per individual animal in descriptive terms, including location(s), size, shape, color, consistency, and number as appropriate.

### 2.9. Microscopic Findings

For the purpose of reviewing the microscopic findings, the term mononuclear cells encompasses three inflammatory cell types, including lymphocytes, plasma cells, and macrophages (Table 1).

### 2.10. Tissue Collection and Preservation

Tissues were collected for potential future virological analysis, as indicated, and placed into tubes and stored at −60 to −90 °C. Tissues were also collected and fixed by immersion in labeled containers of 10% neutral buffered formalin (Valtech, Baltimore, MD, USA). All formalin-fixed tissues remained in the BSL-3 suite until the method for formalin inactivation of SARS-CoV-2-infected tissue was reviewed and deemed sufficient for the complete inactivation of SARS-CoV-2 in tissue, at which time the samples were removed from BSL-3 biocontainment. The tissues were transported to the histology laboratory, where they were processed for histology, immunohistochemistry, and molecular pathology analysis. 

### 2.11. Immunohistochemistry

Immunohistochemistry (IHC) was performed using the Dako Envision system (Dako Agilent Pathology Solutions, Agilent, Santa Clara, CA, USA) as described previously [72]. Briefly, after deparaffinization, peroxidase blocking, and antigen retrieval, the sections were covered with a mouse monoclonal anti-SARS-CoV-2 nucleocapsid protein (Sino Biological, Beijing, China) antibody at a dilution of 1:4000 and incubated at RT for 45 min. They were rinsed, and the peroxidase-labeled polymer (secondary antibody) was applied for 30 min. Slides were rinsed, and a brown chromogenic substrate 3,3′ Diaminobenzidine (DAB) solution (Dako Agilent Pathology Solutions, Agilent, Santa Clara, CA, USA) was applied for eight min. The substrate–chromogen solution was rinsed off the slides and counterstained with hematoxylin and eosin (H&E). Slides were rinsed, and the sections were dehydrated, cleared with Xyless, and then cover slipped.

### 2.12. Immunofluorescence Cellular Analysis of Ferret Tissue

Formalin-fixed paraffin-embedded (FFPE) tissue sections were deparaffinized using xylene (Millipore Sigma, St. Louis, MO, USA) and a series of ethanol washes. After 0.1% Sudan black B (Sigma-Aldrich, St. Louis, MO, USA) treatment to eliminate the autofluorescence background, the sections were heated in Tris-EDTA buffer (10 mM Tris base, 1mM EDTA Solution, 0.05% Tween 20, pH 9.0) for 20 min to reverse formaldehyde crosslinks. After rinsing with PBS (pH 7.4), the sections were blocked with PBST (PBS + 0.1% Tween-20) containing 5% normal goat serum overnight at 4 °C. Then, the sections were incubated with primary antibodies: mouse monoclonal anti-E-cadherin antibody (Thermo Fisher Scientific, Waltham, MA, USA; 33–4000) at a 1:100 dilution; mouse monoclonal anti-Pancytokeratin antibody AE1/AE3 (Dako Agilent Pathology Solutions, Agilent, Santa Clara, CA, USA; M351529-2) at a 1:100 dilution; rabbit monoclonal anti-SARS-CoV-2 Spike antibody (40150-T62-COV2, Sino Biological, Beijing, China) at a 1:200 dilution; rabbit polyclonal anti-CD3 antibody (A045229-2, Dako Agilent Pathology Solutions, Agilent, Santa Clara, CA, USA) at a 1:200 dilution; mouse monoclonal anti-MX1 antibody (MABF938, Millipore Sigma, St. Louis, MO, USA) at a 1:200 dilution; rabbit anti-ACE2 (Abcam, Waltham, MA, USA; ab15348) at 1:200 dilution; and/or rabbit anti-Myeloperoxidase (MPO) antibody (Dako Agilent Pathology Solutions, Agilent, Santa Clara, CA, USA; A039829-2) at a 1:200 dilution. After rinses with PBST, the sections were incubated at a 1:500dilution with secondary goat anti-rabbit Alexa Fluor 488 (Thermo Fisher Scientific, Waltham, MA, USA) and goat anti-mouse Alexa Fluor 568 (Thermo Fisher Scientific, Waltham, MA, USA) antibodies for 1 h at RT. Sections were cover slipped using the Vectashield mounting medium with DAPI (Vector Laboratories, Newark, CA, USA). Images were captured on a Zeiss LSM 880 confocal system and processed using Image J software Version 1.53t (National Institutes of Health).

## 3. Results

### 3.1. SARS-CoV-2 Intranasal and Small Particle Aerosol Exposure Delivered Dose 

**Figure 1:**
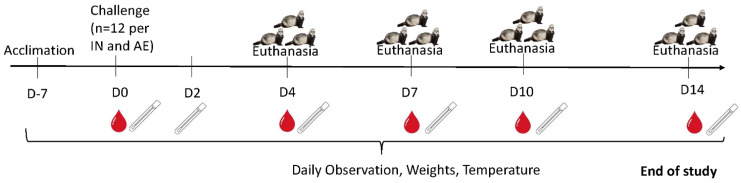
Timeline of study activities. The study in-life for 12 ferrets occurred over a 14-day period with daily observations, weight collections, and temperature monitoring. On the day of challenge (D0), pre-exposure blood collection was performed, and ferrets were exposed to SARS-CoV-2 via IN or AE routes (n = 12 per route). Nasal washes were collected on days 2, 4, 7, 10, and 14, and pharyngeal swabs were collected on days 0, 2, 4, 7, 10, and 14 for analysis of viral load by RT-PCR. On days 4, 7, 10, and 14, three animals from each exposure group were euthanized, and terminal blood collection and necropsy for collection of tissues were conducted. Survival and Clinical Signs of Disease.

We challenged 24 male ferrets weighing 1.59–1.84 kg with SARS-CoV-2 by intranasal (IN) and small particle aerosol (AE) exposure routes. Ferrets in the IN exposure group ranged in age from 5.5 to 6.7 months, and the aerosol (AE) group ranged from 6.0 to 6.1 months. Ferrets challenged IN were exposed to 1.58 × 10^6^ plaque-forming unit (pfu) SARS-CoV-2 as determined by plaque assay back-titration of the challenge material. AE ferrets received doses ranging from 1.24 × 10^5^ to 1.96 × 10^5^ pfu (average = 1.65 × 10^5^ pfu). Intranasal (IN) and aerosol (AE) arms were conducted separately. Three animals from each group were euthanized at 4, 7, 10 and 14 dpe (Figure 1). 

All IN and AE SARS-CoV-2-exposed ferrets received clinical scores of 0 at all time points for natural/provoked behavior, indicating normal behavior. Coughing was observed in only one instance in one ferret (FE23) exposed via the aerosol route. Sneezing or labored breathing was not observed in any ferrets. 

No significant weight loss was observed after IN or small particle AE exposure, and it was not a prominent or consistent feature of either model. A small average drop in weight was observed in both IN- and AE-exposed ferrets at 1 dpe. Overall, weight was more impacted by IN exposure of ferrets. As a group, after 1 dpe, IN-exposed ferrets had a small (an average of approximately 2%) drop in average weight, whereas the AE-exposed ferrets did not (Figure S1A). There was more variability in the percent weight change in the IN-exposed ferrets compared to the AE exposure group. One IN-exposed animal (FE05) had three isolated days where reduced body weight (up to 10%) was observed. Five IN-exposed ferrets had a 4–5% decrease in weight within the first week of exposure, five ferrets lost approximately 2%, and two ferrets did not lose weight. Among the AE-exposed ferrets, 10 animals lost between 2% and 3% of body weight at some point post-exposure. Most of these had a dip in weight at 1 dpe. Two AE-exposed ferrets did not lose weight. 

Overall, there were no trends showing fever or elevated temperature in ferrets exposed to SARS-CoV-2 by either route (Figure S1B). Average temperatures stayed within 1 °C of the baseline for the duration of the study. Baseline values were based on temperatures recorded at day 0 prior to exposure for each animal.

### 3.2. SARS-CoV-2 Detection by RT-PCR

**Figure 2:**
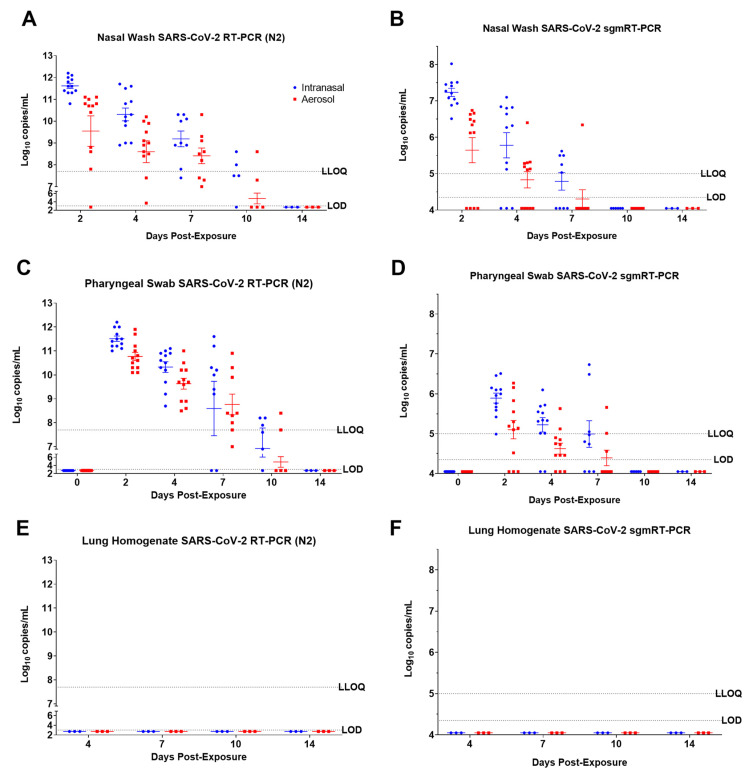
SARS-CoV-2 N2 RNA and subgenomic E RNA in ferret samples. Real-time RT-PCR was used to quantify the amount of total SARS-CoV-2 N2 RNA in nasal swabs (**A**), pharyngeal swabs (**C**), and lung homogenates (**E**), or subgenomic mRNA (sgm) SARS-CoV-2 E RNA in nasal swabs (**B**), pharyngeal swabs (**D**), and lung homogenates (**F**) in ferrets challenged via intranasal or small particle aerosol routes with SARS-CoV-2. (**E**,**F**) results are from terminal samples. Individual data points are plotted with the horizontal line representing the mean and the vertical lines representing the standard error of the mean (SEM). The LLOQ and LOD are represented by dashed lines. Values below the LOD are plotted as ½ LOD.

Pharyngeal swabs were collected from all animals on days 0, 2, 4, 7, 10, and 14, and nasal washes were collected from all animals at 2, 4, 7, 10, and 14 dpe. Three different types of viral genomic RNA (vRNA) detection assays were conducted utilizing nucleoprotein (N2) (Figure 2), glycoprotein (GP) (Figure S2), and E gene-specific (Figure S2) RT-PCR assays. An E gene-specific subgenomic mRNA (sgmRNA) RT-PCR assay was also conducted to evaluate replicating viruses in the samples (Figure 2). All nasal wash samples collected at 2 and 4 dpe, regardless of exposure route, were positive for SARS-CoV-2 vRNA as determined by N2-specific RT-PCR (Figure 2A). Most ferrets were also positive for vRNA at 7 and 10 dpe (Figure 2A and Figure S2A). The highest titers were detected at 2 dpe, with titers steadily decreasing through 10 dpe. SARS-CoV-2 vRNA was not detected in nasal washes collected at 14 dpe for either exposure route (Figure 2A and Figure S2A). All ferrets exposed to SARS-CoV-2 via the IN route were positive for sgmRNA in nasal wash samples collected from at least one time-point, indicating active viral replication (Figure 2B); 10 of 12 AE-exposed ferrets had at least one positive sgmRNA nasal wash sample. Replication was slightly more pronounced in the IN exposure group compared to the AE exposure group. In general, for both IN- and AE-exposed ferrets, sgmRNA titers were highest at 2 dpe and were lower at 4 and 7 dpe (Figure 2B). Ferret nasal wash samples were all below the level of detection for sgmRNA at 10 or 14 dpe (Figure 2B). Among the AE-exposed ferrets, however, sgmRNA titers in nasal wash samples collected from two AE-exposed ferrets remained below the limit of detection at all time points (FE17 and FE24). In conclusion, sgmRNA detection assay results confirm that SARS-CoV-2 establishes a productive infection in the nasal passage following both IN and AE exposure.

Genomic RT-PCR assays for GP (Figure S2C) and N2 (Figure 2C) vRNA were conducted on pharyngeal swab samples for all animals at 0, 2, 4, 7, 10, and 14 dpe. The trends of the pharyngeal swab vRNA data in both IN- and AE-exposed ferrets mirror that of the nasal wash vRNA results, with the highest titers obtained at 2 dpe and titers generally decreased thereafter (Figure 2C). All animals exposed to IN or AE were vRNA-positive for N2 at 2 and 4 dpe (Figure 2C). Most of these samples were also positive at 7 dpe, with all of the AE-exposed ferrets and 7 of 9 IN-exposed ferrets testing positive for N2 vRNA. At 10 dpe, IN-exposed ferrets had greater positivity than AE-exposed ferrets in pharyngeal swab samples using the N2 assay, and the IN-exposed ferrets had a greater portion of animals with positive samples (5 of 6) compared to the AE-exposed ferrets (2 of 6). Similar to the nasal wash results, vRNA was not detected in any ferrets at 14 dpe by either RT-PCR assay. All ferrets, regardless of exposure route, had measurable sgmRNA titers in pharyngeal swabs from at least one time point (Figure 2D). Overall, sgmRNA titers were highest at 2 dpe and were lower at 4 and 7 dpe for both challenge routes (Figure 2D). No pharyngeal swab samples were positive for sgmRNA at 10 or 14 dpe (Figure 2D). 

vRNA and sgmRNA titers in lung homogenates (Figure 2E,F) and serum (Figure S3) remained below the limit of detection for each assay at all time points for both IN and AE exposure routes.

### 3.3. Humoral Immune Response to SARS-CoV-2

**Figure 3:**
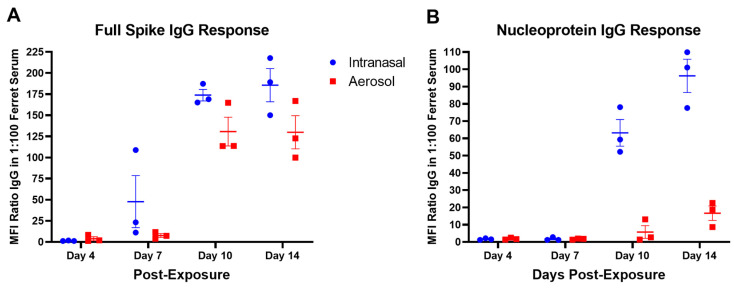
SARS-CoV-2 humoral response in intranasal- and aerosol-exposed ferrets. Serum was collected on days 4, 7, 10, and 14 post-exposure from ferrets exposed to SARS-CoV-2 by the intranasal or aerosol route and evaluated by MAGPIX for the IgG response to SARS-CoV-2 full spike antigen (**A**) or nucleoprotein (**B**). Positive IgG responses by MAGPIX were evaluated in 1:100 ferret serum to SARS-CoV-2 full spike protein (**A**) or nucleoprotein (**B**). Positive results were indicated as ratio values > 4. Individual data points are plotted with the horizontal line representing the mean and the vertical lines representing the SEM.

To evaluate seroconversion, a MAGPIX assay was performed using beads coupled with either full-length spike (S) or nucleoprotein (NP) to assess virus-specific IgG in serum collected at indicated times post-exposure. For IN-challenged ferrets, there was no significant detection of S-specific IgG at 4 dpe. For AE-challenged ferrets, S-specific IgG was detectable in the serum of one animal (FE17) at 4 dpe (Figure 3A). For both IN and AE ferrets, S-specific IgG was detected in the serum at 7 dpe, with levels being higher for animals exposed via the IN route (Figure 3A). The IgG response to S became more pronounced through 14 dpe. At 10 dpe, NP-specific IgG was detectable in both IN and AE challenged ferrets and remained detectable through 14 dpe (Figure 3B). Overall, S-specific IgG titers were higher relative to NP-specific IgG titers and were detectable earlier post-exposure. S-specific titers, as compared to IgG response, were statistically significant by a Pearson correlation test in both IN- and AE-exposed ferrets (P = 0.0057 IN, P = 0.0129 AE) with an R^2^ value of 0.9886 and 0.9743, respectively. 

### 3.4. SARS-CoV-2 Induced Pathology

**Figure 4:**
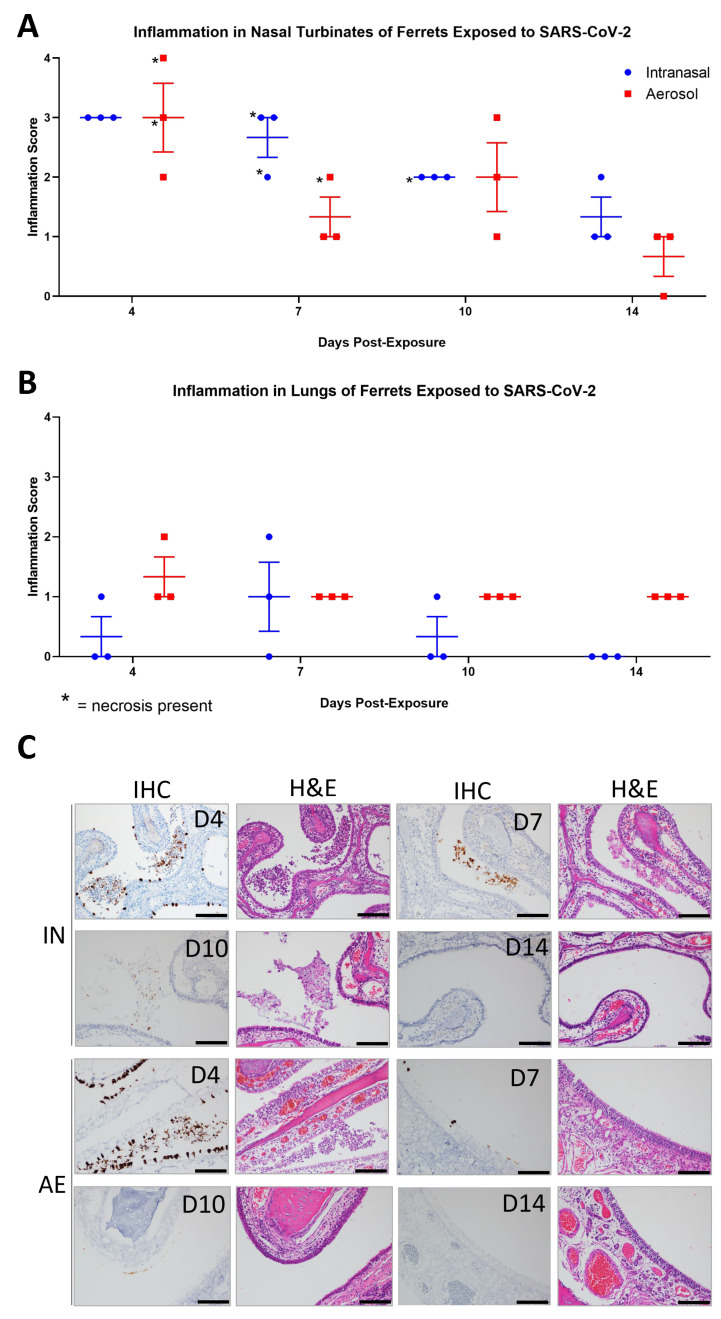
Immunopathology of SARS-CoV-2 in the nasal turbinates and lungs of ferrets. Nasal turbinates and lungs from ferrets infected IN or AE with SARS-CoV-2 were analyzed at days 4, 7, 10, and 14 post-exposure by hematoxylin and eosin (H&E) staining and anti-SARS-CoV-2 immunostaining. H&E slides of nasal turbinates and lungs were evaluated and scored for inflammation (**A**,**B**), and individual data points were plotted with the horizontal line representing the mean and the vertical lines representing the SEM. For immunohistochemistry (IHC) analysis (**C**), SARS-CoV-2 NP immunopositivity appears as a brown precipitate, nuclei are stained by hematoxylin (blue). and the corresponding H&E-stained images are shown to the right of each IHC image. Scale bar = 100 µm.

No significant differences in pathology were noted when comparing the nasal turbinates of IN and AE groups. More aerosol-challenged animals demonstrated lung pathology than those challenged by the IN route; however, with almost all lesions not rising above minimal, the significance of this observation is unclear. The most common microscopic findings in ferrets for both IN and AE groups consisted of inflammation in the nasal turbinates (23/24 animals; 96%) composed of neutrophils and mononuclear cells (Figure 4A). Six animals had necrosis of the mucosa consisting of single-cell death and loss. There was no full-thickness necrosis or ulceration of the nasal turbinates noted in any of the animals. Vacuolation of the mucosal epithelial cells and congestion were other findings noted in the nasal turbinates. Inflammation in the lungs was seen in 17 of 24 animals (5 of 12 IN and 12 of 12 AE, 42% and 100%, respectively), most commonly around affected blood vessels and within the alveolar lumen (Figure 4B). Inflammation consisted of neutrophils and macrophages with few lymphocytes and plasma cells. Rarely were syncytial cells present. Necrosis was not a prominent feature observed in the lungs of these animals (Figure 4B). Other microscopic changes included congestion, osseous metaplasia, and the presence of inflammatory infiltrates. Lymphoid hyperplasia was observed in the mesenteric lymph node and tracheobronchial lymph node. Other reported abnormalities included osseous metaplasia in the lung, vacuolated bronchial epithelium, inflammatory infiltrates in various organs, and draining hemorrhage in the tracheobronchial lymph node were considered incidental changes and not contribute to SARS-CoV-2 exposure.

In IN-exposed ferrets, SARS-CoV-2 NP was detected in the epithelial cell lining of the nasal turbinates and in sloughed cells in nasal cavities in 3 of 3 ferrets at 4 dpe, 3 of 3 ferrets at 7 dpe, 1 of 3 ferrets at 10 dpe, and 0 of 3 ferrets at 14 dpe (Figure 4C). Similarly, in AE-exposed ferrets, SARS-CoV-2 NP was detected in the epithelial cell lining of the nasal turbinates and in sloughed cells in nasal cavities in 3 of 3 ferrets at 4 dpe, 2 of 3 ferrets at 7 dpe, 0 of 3 ferrets at 10 dpe, and 0 of 3 ferrets at 14 dpe. The number of NP-positive cells detected in the nasal turbinates decreased significantly at time points more distal to exposure for both IN- and AE-exposed ferrets (Figure 4C). Animal FE08, euthanized at 10 dpe, had rare nasal turbinate epithelial cells that were positive for viral antigen. NP was detected in individual epithelial cells and did not exceed 25% of the cells in the tissue section. SARS-CoV-2 NP-positive cells were undetectable in the trachea, olfactory bulbs, lung, tracheobronchial lymph nodes, and kidney of all IN- and AE-exposed ferrets at 4, 7, 10, and 14 dpe. 

### 3.5. ACE2 Expression in Ferrets Exposed to SARS-CoV-2

**Figure 5:**
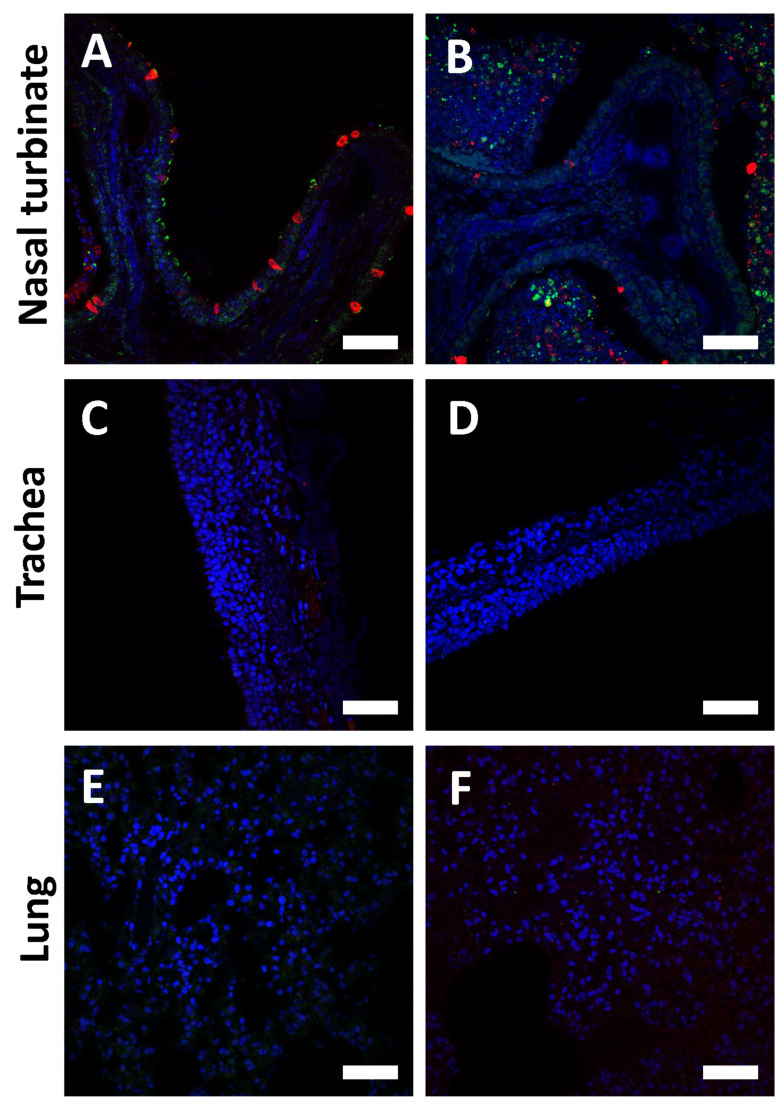
Immunofluorescence images of nasal turbinates, trachea, and lung at 4 dpe from ferrets infected by the IN route with SARS-CoV-2. Immunofluorescence analysis of nasal turbinate (**A**,**B**), trachea (**C**,**D**), and lung (**E**,**F**) from a ferret exposed to SARS-CoV-2 by the IN route and euthanized 4 dpe. Samples were stained for SARS-CoV-2 (red), ACE2 (green), and DAPI nuclear stain (blue). Scale bar T = 50 µm.

To investigate the lack of substantial SARS-CoV-2 infection in the lower respiratory tract of ferrets, we assessed the expression profile of ACE2 across the entirety of the respiratory tract. Immunofluorescence staining of nasal turbinate, trachea, and lung tissue from ferrets exposed to SARS-CoV-2 revealed that SARS-CoV-2 NP and ACE2 detection was abundant in the epithelium of nasal turbinate but undetectable in the trachea and lungs (Figure 5). ACE2 was predominantly detected in the motile cilia of the nasal airway and sloughed cells in the nasal cavities. 

### 3.6. Phenotyping SARS-CoV-2 Positive Cells and Immune Cell Infiltrates

**Figure 6:**
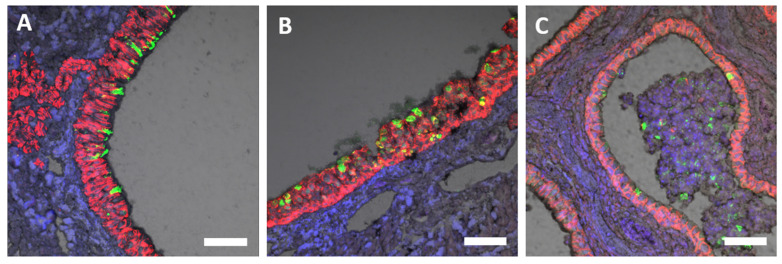
Detection of SARS-CoV-2 spike protein in the nasal turbinates at 4 dpe by immunofluorescence staining. Nasal turbinates collected from SARS-CoV-2 IN-exposed ferrets on day 4 post-exposure were formalin-fixed and immunostained with antibodies to (**A**–**C**) SARS-CoV-2 spike protein (green) and (**A**,**C**) e-cadherin+ (red) or (**B**) or pan-cytokeratin+ (red). Nuclei were stained blue with 4′,6-diamidino-2-phenylindole (DAPI). Grey is the channel of differential interference contrast (DIC) image of the tissues stained. Scale bar = 50 µm (**A**–**C**).

**Figure 7:**
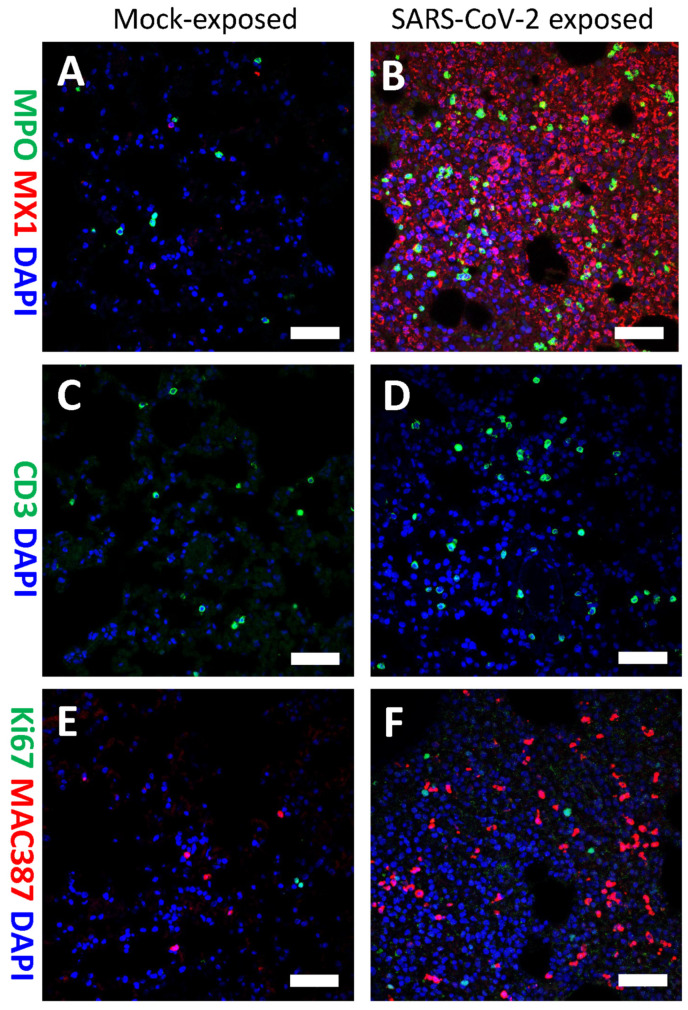
Detection of inflammatory infiltrates in the lungs at 4 dpe by immunofluorescence staining. Lungs collected from unexposed control ferrets (**A**,**C**,**E**) or SARS-CoV-2 IN-exposed ferrets (**B**,**D**,**F**) on day 4 post-exposure were formalin-fixed and immunostained with antibodies to (**A**,**B**) myeloperoxidase (MPO)+ polymorphonuclear cells (neutrophils, eosinophils, and basophils) (green) and (**A**,**B**) type 1 interferon-induced GTP-binding protein MX1 (red), or (C,D) CD3 T cells (green), or (**E**,**F**) Ki67+ for proliferating cells (green) and (**E**,**F**) MAC387+ for macrophages (red). Nuclei were stained blue with 4′,6-diamidino-2-phenylindole (DAPI). Scale bar = 50 µM (**A**–**F**).

To identify cellular targets of SARS-CoV-2 infection in the upper and lower respiratory tract of ferrets, tissues collected at various time points post-exposure were fixed and immunostained with SARS-CoV-2-specific and cell-type-specific antibodies. SARS-CoV-2 spike protein was predominantly detected in e-cadherin^+^ or pan-cytokeratin^+^ epithelial cells lining the nasal turbinates, as well as in sloughed cells within the nasal turbinates (Figure 6). Compared to historical unexposed ferret lung tissues, an abundance of immune cell infiltrates, including myeloperoxidase (MPO)^+^ polymorphonuclear cells (neutrophils, eosinophils, and basophils), were detected in the lung of animal FE02 at 4 dpe (Figure 7A,B). CD3^+^ T-cells and Ki67^+^ cells (a marker for proliferation) were also found in lung tissue from IN-exposed animals (Figure 7C–F). MAC387^+^ macrophages were also more abundant in the lungs of IN-exposed animal FE02 at 4 dpe, relative to mock exposed ferrets (Figure 7E,F). Additionally, the presence of type 1 interferon-induced GTP-binding protein Mx1 in the lung at 4 dpe suggests type I interferon might play a role in limiting SARS-CoV-2 infection of the lower respiratory tract (Figure 7B).

### 3.7. Environmental Swabs

To assess the potential for fomite transmission between cage mates and the utility of ferrets in transmission studies, environmental samples within the cage were collected at various time points post-exposure. Genomic RT-PCR assays for GP and N2 viral gene expression were conducted on swabs collected from the nesting area and feeder rim inside the cage enclosure for each ferret exposed to SARS-CoV-2 by the intranasal (IN) route. Samples were collected on day 0 (prior to the challenge) and 2, 3, 7, and 10 dpe. All day 0 samples were negative, as expected (Figure S4). Interestingly, most swab samples collected from the feeder rim and the nesting area were positive at 2, 4, and 7 dpe by at least one genomic RT-PCR assay (Figure S4). The amount of environmental SARS-CoV-2 detected diminished by 10 dpe and was more prevalent on the feeder rim inside the cage than in the nesting area within the cage (Figure S4). These results suggest that if ferret enclosures were configured in an adjoining fashion, transmission between adjacent ferrets may be possible.

## 4. Discussion

In this study, we exposed ferrets to approximately 10^5^ pfu SARS-CoV-2 (WA-1/2020) by either the IN or small particle (~3 µm) AE route. As with studies reported by others, ferrets in our study did not develop significant clinical signs of disease following exposure to SARS-CoV-2, as evidenced by a lack of consistent body temperature elevation, remarkable weight loss, or mortality [15,31,32,34,35,51,52,62]. However, ferrets were productively infected with SARS-CoV-2 via both IN and small particle AE routes, as demonstrated by the presence of subgenomic viral RNA and positive SARS-CoV-2 staining by immunohistochemistry and immunofluorescence. In addition, a SARS-CoV-2-specific IgG response to SARS-CoV-2 spike and nucleoprotein was measured in AE- and IN-exposed ferrets. The prevalence of neutralizing IgG antibodies and spike-specific antibodies following SARS-CoV-2 infection of ferrets has also been demonstrated by other groups [15,34]. These observations were consistent in ferrets exposed to SARS-CoV-2 via either the IN or AE routes [73]. The mild disease progression and clinical similarities following IN and large particle AE exposure in ferrets have also been reported by other laboratories [24,25,26,28,29,32,34,35,39,47,51,62,74].

Interestingly, SARS-CoV-2 infection did not result in detectable viral replication in the lower respiratory tract, and little to no viral genomic material was recovered from lung homogenates (Material not intended for publication, Zumbrun, USAMRIID, Frederick, MD, archived data, 2023). The findings by Ryan et al. similarly showed that IN SARS-CoV-2 exposure of ferrets resulted in detectable yet unquantifiable levels of viral RNA in the lungs of ferrets at 3 dpe [34]. These findings correlate with the lack of positive immunohistochemistry staining in lung samples across all time points and are also supported by previous reports that observed little to no evidence of productive SARS-CoV-2 infection in the lungs of ferrets [33,34,35,51]. The presence or absence of viral replication could be due to differing expression levels of ferret ACE2 in the upper and lower airways of ferrets, which, along with mink, shares 94% homology with human ACE2 [9,75]. Reports on ACE2 distribution in ferret lungs differ. Van den Brand et al. demonstrated the presence of ACE2 in type-1 pneumocytes lining the alveolar surface and bronchial goblet cells and the presence of SARS-CoV-1, which also utilizes the ACE2 receptors for entry, in the lungs of ferrets [20]. In contrast, Lean et al. demonstrated the presence of ACE2 in both the upper and lower respiratory tracts of mink, which can have severe lung pathology resulting from SARS-CoV-2 infection, but observed ACE2 expression only in the upper respiratory tract in ferrets [75]. Here, we have demonstrated that ACE2 was prevalent in the epithelium of nasal turbinate but undetectable in the trachea and lungs. Thus, it is likely that the absence of detectable SARS-CoV-2 replication in the lower respiratory tract of ferrets is due, at least in part, to the absence of ACE2-expressing cells there.

Ferrets exposed to SARS-CoV-2 by the IN route had slightly greater severity of pathology in tissues of the upper respiratory tract as compared to the AE exposure group. Also, slightly more virus was detected in the nasal washes and pharyngeal swabs of IN-exposed ferrets as compared to the AE exposure group. Differences in pathology severity and SARS-CoV-2 titer by RT-PCR may be due to a log difference in exposure between IN and AE routes. Additionally, more of the IN-administered viral inoculum may have come into contact with susceptible tissue of the upper respiratory tract than the small particle AE inoculum, which also would have been dispersed across the lower respiratory tract. 

In this study, we observed significant increases in immune cell infiltration by polymorphonuclear cells (neutrophils, basophils, and eosinophils) and macrophages, a slight increase in CD3+ T cell infiltration, and a significant upregulation of type 1 interferon-induced GTP-binding MX1 protein at 4 dpe in lungs of IN inoculated ferrets as compared to controls. Immune cell infiltration into the lower respiratory tract may contribute to the lack of robust viral replication and overt clinical disease signs, although the lack of ACE2 expression in the lower respiratory tract is likely playing a more significant role. Inflammatory cell infiltration in the lungs, particularly neutrophils and macrophages, was also observed by other groups at 3, 5, and 7 dpe in ferrets IN-exposed to SARS-CoV-2 [15,34]. 

RT-PCR analysis of environmental swab samples collected from the IN group demonstrates that the virus was shed into the surrounding environment and suggests the ferret model may be suitable for transmission studies. Direct and indirect transmission between ferrets in conjoined cage configurations, as well as with distances of up to 1 m between cages, has been reported [15,49,51]. Naïve ferrets in direct contact with SARS-CoV-2 infected animals were positive within the nare as early as 2 dpe and were positive by RT-PCR for replicating virus 8 days after exposure, which may accurately represent in vivo viral incubation times [15,51]. Leveraging the ferret model to better understand SARS-CoV-2 transmission could aid in our overall comprehension of viral transmission during the COVID-19 pandemic as well as the potential impact SARS-CoV-2 may have on the agricultural industry, including mink farms [76]. 

IN or small particle AE SARS-CoV-2 exposure ferret models may be useful for assessing SARS-CoV-2 vaccine and therapeutic candidates [24,25,29,41,42], viral transmission [28,29], and the impacts of viral variants and host factors on disease severity [27] [77,78]. Frequent nasal wash samples with genomic and subgenomic RNA, as well as pathology in the upper respiratory tissues at early time points, are ideal primary endpoints for the ferret model. The SARS-CoV-2 IN ferret model demonstrated positive findings, such as enhanced viral replication and pathology in the upper respiratory tract, that overall was of a slightly higher magnitude than that of ferrets exposed to small particle AE. While the AE model may more accurately mimic natural transmission events and infection [4,44,53,54,55,56], the IN exposure route is more easily executed, and a greater amount of virus is delivered directly to the target tissue of the upper respiratory tract. The IN and AE SARS-CoV-2 exposure models in ferrets can be used to provide valuable preclinical data on areas such as viral fitness, transmission dynamics, and efficacy of therapeutic and vaccine candidates to public health practitioners, who can then further evaluate and validate the findings in human clinical trials and epidemiological studies.

## Figures and Tables

**Table 1:** Definition of microscopic severity.

Minimal	Change Is Present in Approximately Less than 10% of the Entire Section or Less than 10% of the Cells
Mild	Change is present in approximately 11% to25% of the entire section or cells
Moderate	Change is present in approximately 26% to 50% of the entire section or cells
Marked	Change is present in approximately 51% to 79% of the entire section or cells
Severe	Change is present in approximately >80% of the entire section or cells

## Data Availability

The data presented in this study are available on request from the corresponding author and with permission from USAMRIID.

## Supplementary Materials

The following supporting information can be downloaded at: https://www.mdpi.com/article/10.3390/v15122341/s1.
