# Supplementary material for: SARS-CoV-2 Aerosol and Intranasal Exposure Models in Ferrets
Source: Viruses. 2023 Nov 29;15(12):2341. doi: 10.3390/v15122341 (PMC10747480; doi:10.3390/v15122341)
Supplement: Supplementary file 1 [file viruses-15-02341-s001.zip › viruses-2728024-supplementary.pdf]

Supplemental Information

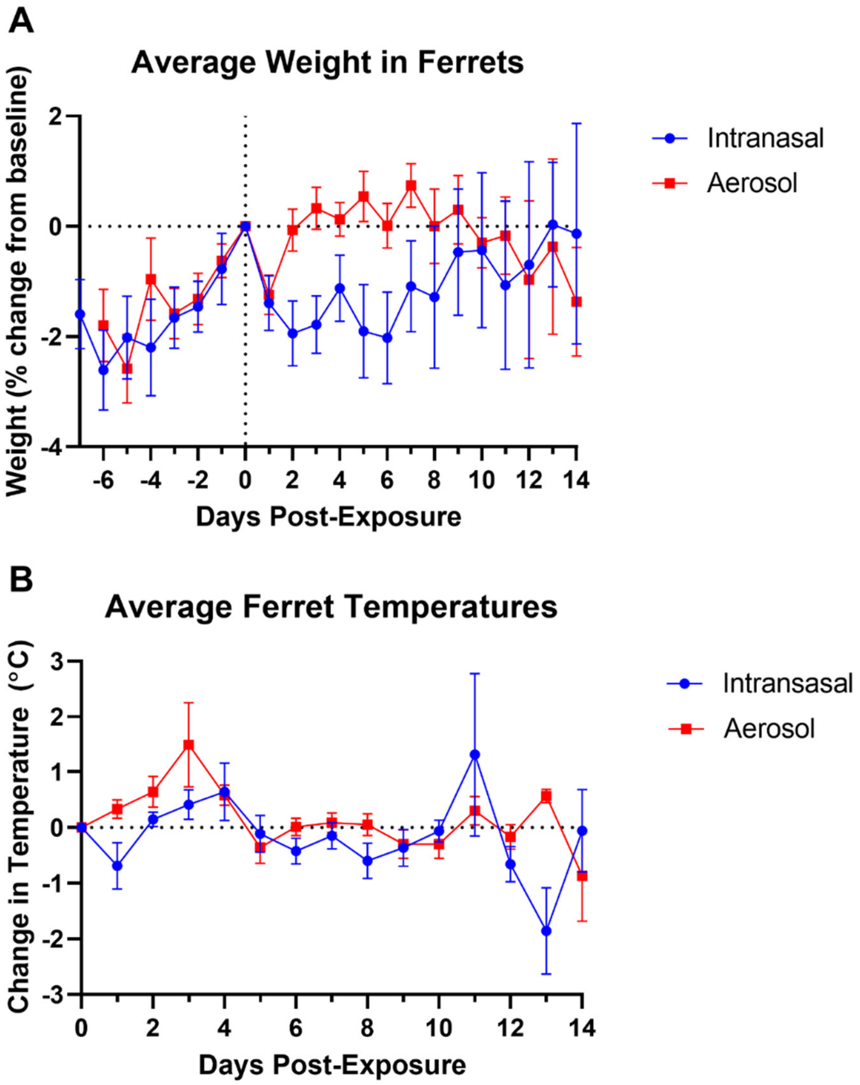

S1 Fig. Average change in body weight (A) and temperature (B) after SARS-CoV-2 exposure in ferrets.

Ferrets were exposed to SARS-CoV-2 by the IN or AE route and body weight and temperatures were monitored daily in each ferret. The mean and SEM are plotted.

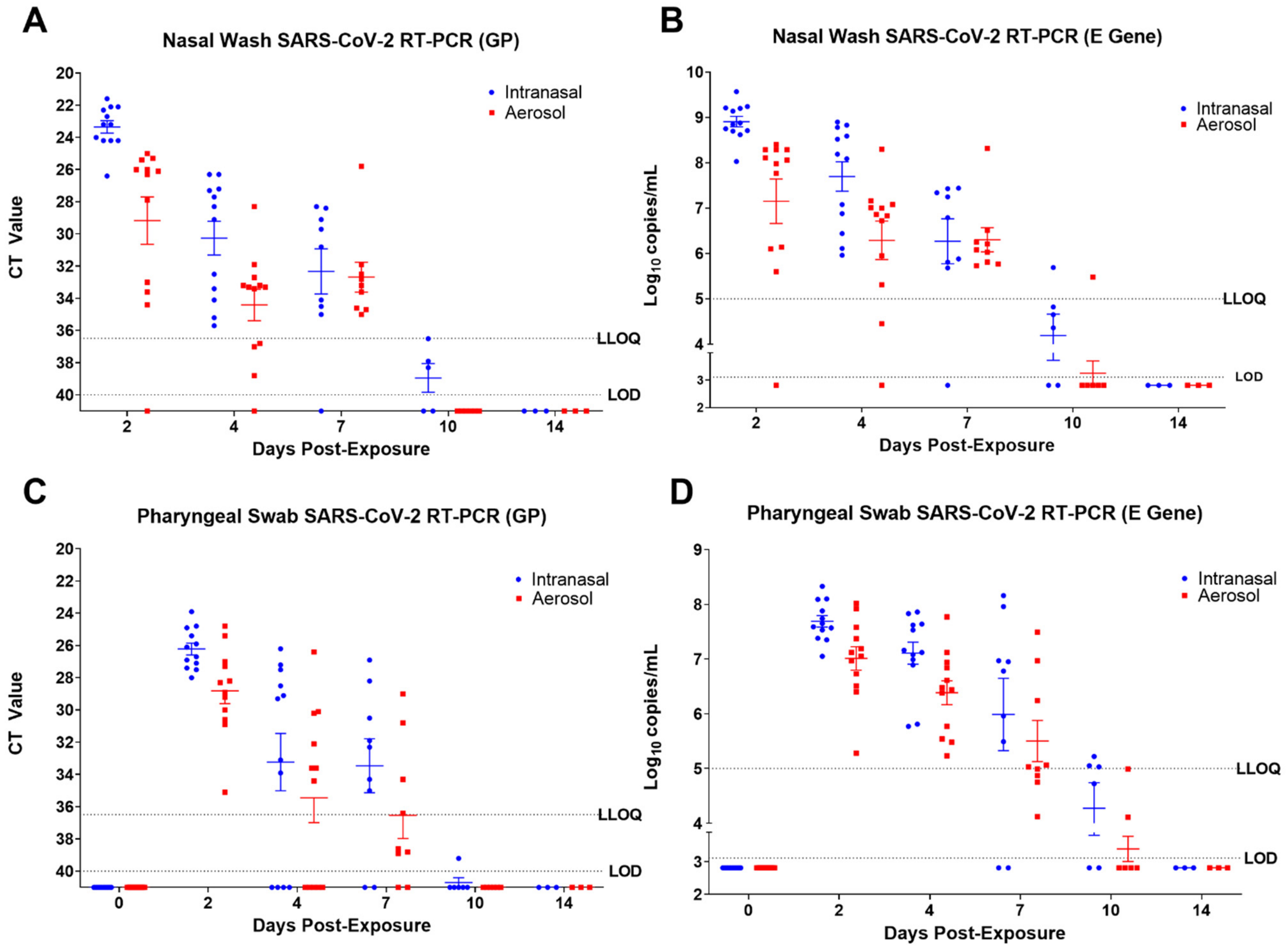

S2 Fig. SARS-CoV-2 GP RNA and subgenomic E gene RNA in ferret samples. Nasal and pharyngeal swabs were collected from ferrets exposed to SARS-CoV-2 by IN or AE route. Real-time RT-PCR was used to quantify the amount of total SARS-CoV-2 GP RNA in nasal swabs (A) and pharyngeal swabs (C) or E RNA in nasal swabs (B) and pharyngeal swabs (D) in intranasal or small particle aerosol challenged ferrets. Individual datapoints are plotted with the horizontal line representing the mean and the vertical lines representing the SEM. The LLOQ and LOD are represented by dashed lines. Values below the LOD are plotted as CT=41 (A and C) or ½ LOD (B and D).

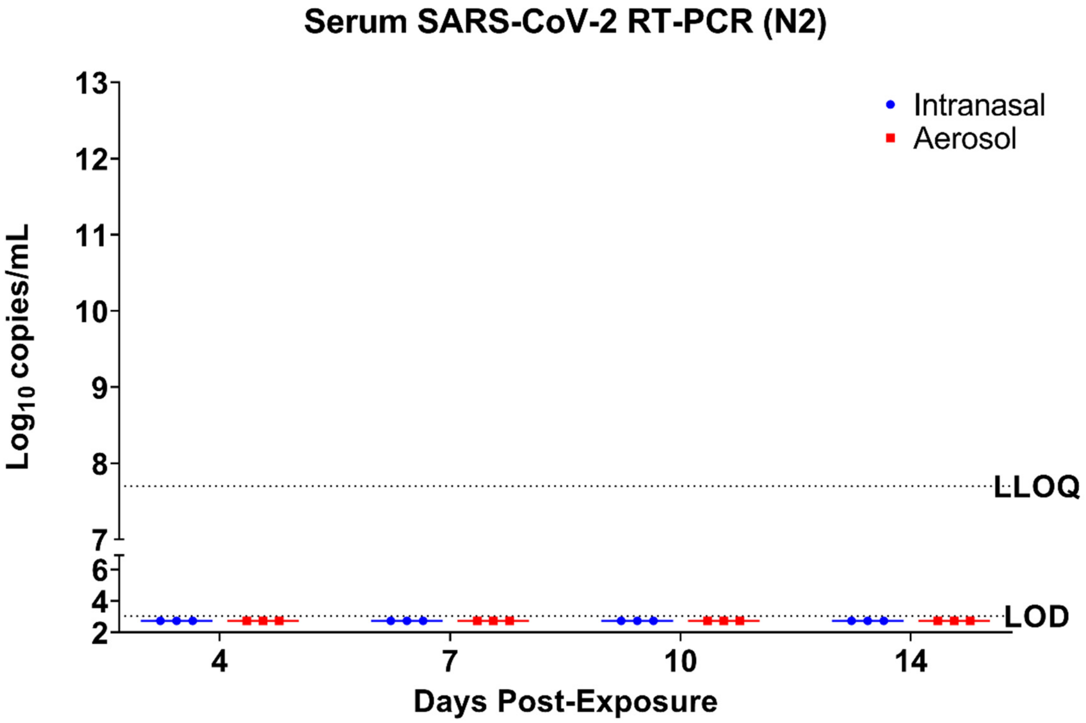

S3 Fig. SARS-CoV-2 N2 RNA in ferret serum samples. Real-time RT-PCR was used to quantify the amount of total SARS-CoV-2 N2 RNA in serum in ferrets challenged by intranasal or small particle aerosol routes with SARS-CoV-2. Results are from terminal samples. Individual datapoints are plotted with the horizontal line representing the mean and the vertical lines representing the standard error of the mean (SEM). The LLOQ and LOD are represented by dashed lines. Values below the LOD are plotted as ½ LOD.

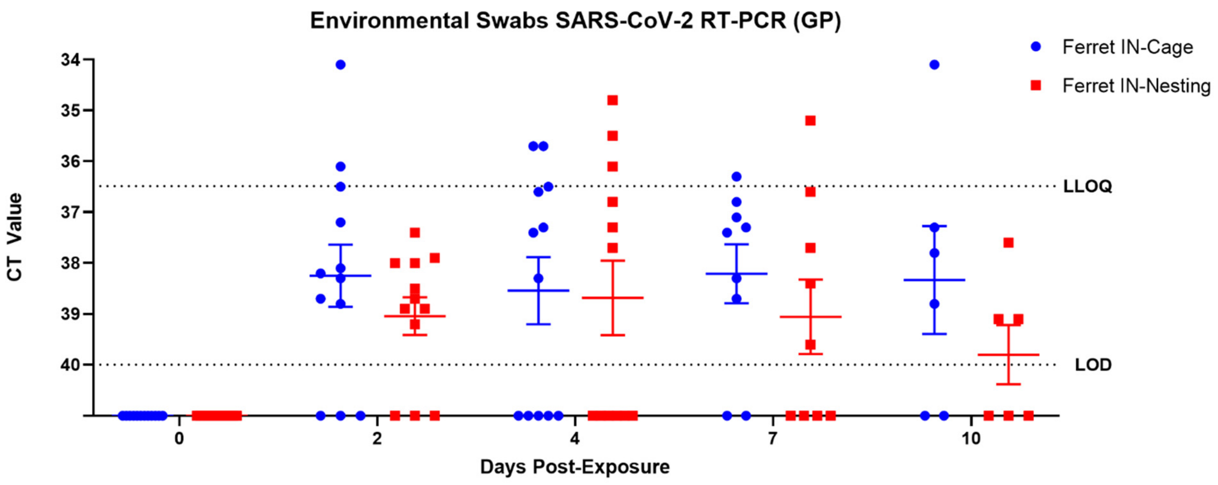

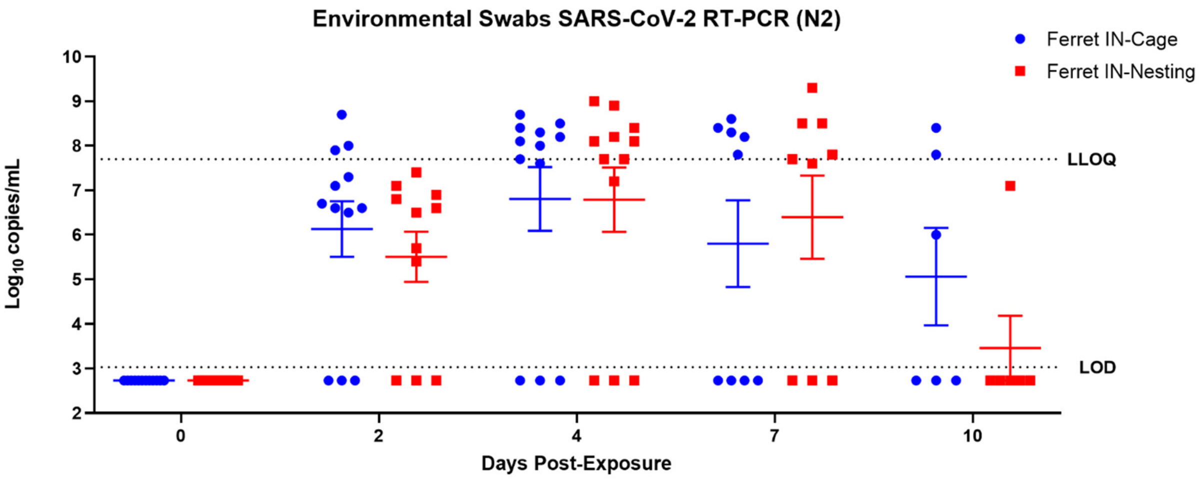

S4 Fig. Environmenal swabs from the nesting box and the feeding containers of ferrets exposed by the IN route to SARS-CoV-2. Ferrets were exposed by the IN route to SARS-CoV-2 and on days 0 (before infection), 2, 4, 7 and 10 the inside of the nesting box and the feeding container (which is placed just outside the cage) were swabbed and placed in media. Real-time RT-PCR was conducted for detection of SARS-CoV-2 glycoprotein (GP) (A) and N2 (B) RNA from nesting and external feeding container swabs. Individual datapoints are plotted with the horizontal line representing the mean and the vertical lines representing the SEM. The LLOQ and LOD are represented by dashed lines. Values below the LOD are plotted as CT=41 (top panal) or ½ LOD (bottom panal).
